# Supplementary material for: COVID-19 Diagnosis: A Comprehensive Review of the RT-qPCR Method for Detection of SARS-CoV-2
Source: Diagnostics (Basel). 2022 Jun 20;12(6):1503. doi: 10.3390/diagnostics12061503 (PMC9221722; doi:10.3390/diagnostics12061503)
Supplement: Supplementary file 1 [file diagnostics-12-01503-s001.zip › diagnostics-1712849-supplementary.pdf]

Table S1. Overview of RT-PCR kits for the diagnosis of COVID-19, approved by FDA under an emergency use authorization (EUA).

S. No.	Name of Kit	Manufacturer	Target Genes	Specimens Used
1.	FOSUN COVID-19 RT-PCR detection kit	Fosun Pharma USA Inc.	E, N and ORF1ab genes	AN, MT, NP, OP swabs, sputum, lower respiratory tract aspirates, BAL, nasal, NP wash/aspirate
2.	TRUPCR SARS-CoV-2 RT-qPCR kit	3B BlackBio Biotech	E, N and RdRP genes	NP, OP, AN, MT swabs, NP aspirates/washes or nasal aspirates, BAL
3.	COVID-19 RT-PCR Test	Laboratory Corporation of America (LabCorp)	N1, N2, and N3 genes	Nasal, NP, OP swabs, Sputum, BAL, Nasal swabs
4.	CRL Rapid Response	Clinical Reference Laboratory, Inc.	RdRp gene	Saliva
5.	OSUWMC COVID-19 RT-PCR test	The Ohio State University Wexner Medical Center	N1 and N2 genes	Nasal, OP, NP swabs
6.	Omni COVID-19 Assay by RT-PCR	Omnipathology Solutions Medical Corporation	N1 and N2 genes	NP, OP, AN, MT swabs, NP wash/aspirate, nasal aspirate, BAL
7.	TaqPath COVID-19 Combo Kit	Thermo Fisher Scientific	S and N genes	NP swab, NP aspirate, BAL
8.	Lilly SARS-CoV-2 Assay	Eli Lilly and Company	N1 and N2 genes	NP, OP, AN, MT swabs, nasal wash/aspirate, BAL
9.	SNL-NM 2019 nCoV Real-Time RT-PCR Diagnostic Assay	Sandia National Laboratories	N gene	NP, OP, AN, MT swabs, nasal wash/aspirate, BAL
10.	CRSP SARS-CoV-2 Real-time Reverse Transcriptase (RT)- PCR Diagnostic Assay	Clinical Research Sequencing Platform (CRSP), LLC at the Broad Institute of MIT and Harvard	N1 and N2 genes	NP, OP, AN, MT swabs, nasal, NP wash/aspirate, BAL
11.	LabGunTM COVID-19 RT-PCR Kit	LabGenomics Co., Ltd.	E and RdRp genes	NP, OP, AN, MT swabs, nasal, NP wash/aspirate, sputum
12.	Real-Time Fluorescent RT-PCR Kit for Detecting SARS-2019-nCoV	BGI Genomics Co. Ltd	ORF1ab gene	OP, NP, AN, MT, nasal swabs, nasal wash/aspirate, BAL
13.	AvellinoCoV2 test	Avellino Lab USA, Inc.	N1 and N3 genes	NP, OP swabs

14.	Alinity m SARS-CoV-2 assay	Abbott Molecular Inc.	RdRp and N genes	NP, OP swabs, BAL
15.	1COPY COVID-19 qPCR multi kit	1DROP INC	E and RdRp genes	NP, OP, AN, MT, swabs, nasal, NP wash/aspirate
16.	Laboratorio ClinicoToledo SARS-CoV-2 Assay	Laboratorio Clinico Toledo	N and E genes	Nasal, NP, MT, OP, BAL swabs
17.	Inform Diagnostics SARS-CoV-2 RT-PCR Assay	Inform Diagnostics, Inc.	N1 and N2 genes	NP, OP, AN, MT swabs, NP wash/aspirate, nasal/aspirates, BAL
18.	Acupath COVID-19 Real-Time (RT-PCR) Assay	Acupath Laboratories, Inc.	ORF1ab, N, and S genes	NP swab, NP aspirate, BAL
19.	Gene By Gene SARS-CoV-2 Detection Test	Gene By Gene	N1 and N2 genes	NP, nasal swabs
20.	Alinity m Resp-4-Plex	Abbott Molecular	RdRp and N genes and Influenza A & B and RSH	NP swabs
21.	Abbott RealTime SARS-CoV-2 assay	Abbott Molecular	RdRp and N genes	NP, OP, nasal swabs
22.	DTPM COVID-19 RT-PCR test	Tide Laboratories, LLC	N gene	NP, OP, MT swabs
23.	MassARRAY® SARS-CoV-2 Panel	Agena Bioscience, Inc.	N genes, ORF-1 and ORF-1ab gene	NP swabs
24.	RealStar® SARS-CoV-2 RT-PCR Kit U.S.	Altona Diagnostics GmbH	E and S gene	NP, OP, AN, MT, nasal swabs, nasal wash/aspirate
25.	BioCode® SARS-CoV-2 Assay	Applied BioCode Inc	N gene	NP, OP, nasal swabs, BAL
26.	Linea™ COVID-19 Real-Time PCR Assay Kit assay kit	Applied DNA Sciences	S gene	Nasal, NP, OP, AN, MT swabs, nasal, NP wash/aspirate
27.	iAMP® COVID-19 Detection Kit	Atila BioSystems, Inc.	N gene and the ORF-1ab gene	NP, OP swabs
28.	BD SARS-CoV-2 Reagents for BD MAX™ System	Becton, Dickinson and Company	N1 and N2 genes	Nasal, NP, OP swabs
29.	BD SARS-CoV-2/Flu Reagents	Becton, Dickinson and Company	N1 and N2 genes and Influenza A /Influenza B	NP, AN swabs
30.	BioCore 2019-nCoV Real Time PCR Kit	BioCore Co. Ltd.	N and RdRp genes	OP, NP, AN, MT swabs, Nasal, NP wash/aspirate, BAL, sputum

31.	Bio-Speedy® Direct RT-qPCR SARS-CoV-2	Bioeksen R&D Technologies Ltd	ORF1ab gene	NP, OP swabs
32.	BioFire® COVID-19 Test	BioFire Defense, LLC	ORF1ab and ORF8	NP swabs
33.	Biofire Respiratory Panel 2.1 (RP2.1)	BioFire Diagnostics LLC	S and M genes	NP swabs
34.	Biofire Respiratory Panel 2.1-EZ (RP2.1-EZ)	BioFire Diagnostics LLC	S gene, M gene, Influenza A and others	NP swabs
35.	BioGX Xfree COVID-19 Direct RT-PCR	BioGX, Inc.	N gene	NP, AN, MT, OP swabs or wash
36.	Influenza SARS-CoV-2 (Flu SC2) Multiplex Assay	Centers for Disease Control and Prevention (CDC)	N gene	NP, OP, nasal swabs, BAL, lower respiratory tract aspirates, NP wash/aspirate, nasal aspirate.
37.	Biomeme SARS-CoV-2 Real-Time RT PCR	Biomeme Inc	ORF1ab and S gene	NP, nasal, OP swab, NP wash/aspirate, nasal aspirate
38.	Biomeme SARS-CoV-2 Go-Strips	Biomeme Inc.	ORF1ab and S gene	Nasal, NP, OP swab, MT swab
39.	BMC-CReM COVID-19 Test	Boston Medical Center	N1 and N2 genes	NP, OP, AN, MT swabs, nasal, NP wash/aspirate, BAL
40.	SARS-CoV-2 RNA DETECTR Assay	UCSF Health Clinical Laboratories, UCSF Clinical Labs	N gene	NP, OP, AN, MT swabs, nasal, NP wash/aspirate
41.	SARS-COV-2 R-GENE®	BioMérieux SA	N gene and RdRp gene	NP, OP, AN, MT swabs, nasal aspirate/wash, BAL
42.	Bio-Rad SARS-CoV-2 ddPCR Kit	Bio-Rad Laboratories Inc	N1 and N2 genes	NP, AN, MT swabs, nasal, NP wash/aspirate
43.	Reliance SARS-CoV-2 Flu A Flu B RT-PCR Assay Kit	Bio-Rad Laboratories Inc	N genes and Influenza A/ Influenza B	NP, AN swabs
44.	Real-Q 2019-nCoV Detection Kit	BioSewoom, Inc.	E gene and RdRp genes	NP, OP, nasal, MT swabs, tracheal, NP aspirate, BAL
45.	COVID-19 RT-PCR PNA kit	BioTNS	RdRp gene and N genes	NP, OP swabs, AN, MT nasal swabs, BAL, NP wash/aspirates or nasal aspirates
46.	Xpert® Xpress SARS-CoV-2 test	Cepheid	N2 and E genes	NP, OP, nasal, MT swabs, nasal wash/aspirate

47.	Xpert Xpress CoV-2/Flu/RSV plus	Cepheid	RdRp and genes N2 and E and Influenza A/Influenza B/RSV RNA	Nasal, NP swabs
48.	Clear Dx™ SARS-CoV-2 Test	Clear Labs, Inc	Sars-Cov-2 Genome	NP, OP swab, AN, MT swabs, NP wash/aspirate, nasal aspirate, BAL
49.	Clinomics TrioDx RT-PCR COVID-19 Test	Clinomics USA Inc	RdRp gene and N and E genes	NP, OP, MT, AN swabs
50.	Fulgent COVID-19 by RT-PCR test	Fulgent Therapeutics, LLC	N gene	Nasal, NP, OP swabs
51.	LOGIX SMART™ Coronavirus Disease 2019 (COVID-19) Kit	Co-Diagnostics, Inc	RdRp gene	BAL, sputum. NP, OP swabs
52.	Assurance SARS-CoV-2 Panel	Assurance Scientific Laboratories	N1 and N2 genes	Nasal, NP, OP swabs
53.	HDPCR™ SARS-CoV-2 Assay	ChromaCode, Inc.	N1 and N2 genes	NP, OP, AN, MT swabs, nasal aspirate/wash, BAL
54.	MobileDetect-BIO BCC19 Test Kit	DetectaChem LLC	N and E genes	NP, OP, MT, AN swabs
55.	Diagnovital SARS-CoV-2 Real-Time PCR Kit	RTA Laboratories Biological Products Pharmaceutical and Machinery Industry	E and RdRP genes	AN, MT, NP, OP swabs, NP wash/aspirates or nasal aspirates, BAL
56.	QuantiVirus SARS-CoV-2 Test kit	DiaCarta, Inc	N, ORF1ab and E genes	Nasal, MT, NP, and OP swabs
57.	QuantiVirus SARS-CoV-2 Mulitplex Test kit	DiaCarta, Inc	ORF1ab gene	Nasal, NP, OP swabs, sputum
58.	OraRisk COVID-19 RT-PCR	Access Genetics, LLC	RdRp gene	Nasal, NP swabs
59.	Simplexa™ COVID-19 Direct assay	DiaSorin Molecular LLC	ORF1ab and S genes	NP, nasal swabs, BAL
60.	AMPIPROBE® SARS-CoV-2 Assay kit	Enzo Life Sciences, Inc	N1 and N2 genes	NP, OP, AN, MT swabs, nasal, NP wash/aspirate
61.	EURORealTime SARS-CoV-2	Euroimmun US Inc.	ORF1ab and N genes	MT, NP, OP swabs, BAL
62.	FTD SARS-CoV-2	Fast Track Diagnostics Luxembourg S.á.r.l. (a Siemens Healthineers Company)	ORF1ab and N genes	Nasal, NP, OP swabs, nasal, NP wash/aspirate, BAL

63.	Advanta Dx SARS-CoV-2 RT-PCR Assay	Fluidigm Corporation	N genes	NP swabs
64.	GenePro SARS-CoV-2 Test	Gencurix, Inc.	ORF1ab and E genes	NP, OP, AN, MT swabs, NP wash/ aspirate, nasal aspirate, BAL
65.	NeoPlex COVID-19 Detection Kit	GeneMatrix, Inc.	RdRp and N genes	NP, OP, MT, nasal swabs, sputum, BAL
66.	Boston Heart COVID-19 RT-PCR Test	Boston Heart Diagnostics	N, S and ORF1ab genes	NP, OP, MT, nasal swabs, NP aspirate, BAL
67.	Genetron SARS-CoV-2 RNA Test	Genetron Health (Beijing) Co., Ltd	ORF1ab and N genes	OP, NP, AN, MT swabs
68.	CareStart COVID-19 MDx RT-PCR	Access Bio, Inc.	RdRp and N genes	NP, OP, nasal swabs,
69.	ePlex® SARS-CoV-2 Test	GenMark Diagnostics, Inc.	N gene	NP swabs
70.	GS COVID-19 RT-PCR KIT	GenoSensor LLC	ORF1ab, N and E genes	NP, OP, nasal, MT swabs
71.	Gnomegen COVID-19 RT-Digital PCR Detection Kit	Gnomegen LLC	N1 and N2 genes	Nasal, NP, OP swabs
72.	Aptima SARS-CoV-2 assay	Hologic, Inc.	ORF1ab Region 1 and 2	NP, nasal, MT, OP swabs, nasal, NP wash/aspirate
73.	Panther Fusion SARS-CoV-2 Kit	Hologic, Inc.	ORF1ab, S and N genes	NP, nasal, OP swabs, lower respiratory tract specimens
74.	Aptima SARS-CoV-2/Flu assay	Hologic Inc	ORF1ab Region 1<br>ORF1ab Region 2<br>Influenza A/B	NP, anterior nasal swab
75.	Hymon™ SARS-CoV-2 Test Kit	HymonBio Co. LTD	N and E genes	NP, OP, MT, nasal swabs, BAL
76.	Smart Detect™ SARS-CoV-2 rRT-PCR Kit	InBios International, Inc	ORF1ab, N and E genes	NP, AN, MT swabs
77.	COV-19 IDx assay	Ipsum Diagnostics, LLC	N gene	NP, OP swabs
78.	COVID-19 Coronavirus Real Time PCR Kit	Jiangsu Bioperfectus Technologies Co Ltd	ORF1ab and N genes	NP, OP, AN, MT swabs, nasal aspirates, nasal washes, BAL, sputum
79.	Novel Coronavirus (SARS-CoV-2) Fast Nucleic Acid Detection Kit	Jiangsu CoWin Biotech Co., Ltd.	ORF1ab and N genes	OP swabs
80.	Helix COVID-19 Test	Helix OpCo LLC (dba Helix)	N, S and ORF1ab genes	NP, OP, AN, MT swabs

81.	PowerChek™ 2019-nCoV Real- time PCR Kit	Kogene Biotech Co Ltd	RdRp and E genes	NP, OP, AN, MT swabs, nasal, NP wash/aspirate, BAL, sputum
82.	LabGun™ COVID-19 RT-PCR Kit	LabGenomics	RdRp and N genes	NP, OP, AN, MT, nasal swabs, NP wash/aspirate or nasal aspirate, sputum
83.	ARIES® SARS-CoV-2 Assay Kit	Luminex Corporation	ORF1ab and N genes	NP swabs
84.	NxTAG CoV Extended Panel Assay	Luminex Molecular Diagnostics, Inc.	ORF1ab, N and E genes	NP swabs
85.	LumiraDx SARS-CoV-2 RNA STAR Complete	LumiraDx UK Ltd	ORF1a Gene	Upper respiratory swabs
86.	SARS-CoV-2 Fluorescent PCR Kit	Maccura Biotechnology (USA) LLC	ORF1ab, N and E genes	OP, NP, nasal, MT swabs
87.	SARS-CoV-2 DETECTR Reagent Kit	Mammoth Biosciences, Inc.	RP and N gene	NP, OP, MT, nasal swabs, AN swabs, NP wash/aspirate, nasal aspirate
88.	Revogene SARS-CoV-2 assay	Meridian	N gene	NP, OP, AN, MT, nasal swabs
89.	Accula SARS-Cov-2 Test	Mesa Biotech Inc.	N gene	Nasal swabs
90.	Gravity Diagnostics COVID-19 Assay	Gravity Diagnostics, LLC	N1 and N2 genes	Nasal, NP, OP swabs, BAL
91.	Phosphorus COVID-19 RT-qPCR Test	Phosphorus Diagnostics LLC	N1 and N2 genes	Saliva
92.	NeuMoDx SARS-CoV-2 Assay	NeuMoDx Molecular, Inc.	Nsp2 and N genes	Nasal, NP, OP swabs
93.	Hymon SARS-CoV-2 Test Kit	Dba SpectronRx	N and E genes	Nasal, OP, NP, AN, MT swabs, BAL
94.	NeuMoDx Flu A-B/RSV/SARS-CoV-2 Vantage Assay	NeuMoDx Molecular, Inc.	Nsp2 target and N gene target and Influenza A/B and RSV M genes	NP, AN swabs
95.	Kaira 2019-nCoV Detection Kit	OPTOLANE Technologies, Inc.	RdRp and E gene	NP, OP, AN, and MT, nasal swabs, NP wash/aspirate, nasal aspirate, sputum, BAL
96.	GeneFinder™ COVID-19 Plus RealAmp Kit	OSANG Healthcare Co., Ltd	ORF1ab, N and E genes	NP, OP, nasal, MT swabs, BAL, sputum
97.	OPTI SARS-CoV-2 RT PCR Test	OPTI Medical Systems, Inc	N1 and N2 genes	Nasal, NP, OP swabs, sputum, TA, BAL, nasal, NP aspirate/wash
98.	P23 Labs TaqPath SARS-CoV-2 Assay	P23 Labs, LLC	N, S and ORF1ab genes	OP, NP, AN, MT swabs, nasal, NP wash/aspirate, BAL

99.	DiaPlexQ™ Novel Coronavirus (2019-nCoV) Detection Kit	SolGent Co., Ltd.	ORF1a and N genes	OP, NP, AN, MT swabs, nasal, NP wash/aspirate, BAL, sputum
100.	DetectX-Rv	PathogenDx, Inc.	N genes	NP, OP, MT, AN swabs, nasal aspirates, NP wash/aspirates, BAL
101.	PerkinElmer® New Coronavirus Nucleic Acid Detection Kit	PerkinElmer, Inc.	ORF1ab and N genes	NP, OP swabs
102.	IntelliPlex SARS-CoV-2 Detection Kit	PlexBio Co., Ltd.	RdRp, N and E genes	NP, OP, AN, MT, nasal swabs, NP wash/aspirate, nasal aspirates, BAL
103.	FastPlex Triplex SARS-CoV-2 detection kit	PreciGenome LLC	ORF1 ab and N genes	OP swabs
104.	MD Anderson High-throughput SARS-CoV-2 RT-PCR Assay	University of Texas MD Anderson Cancer Center, Molecular Diagnostics Laboratory	N1 and N2 genes	NP, OP, MT, nasal swabs
105.	COVID-19 genesig® Real-Time PCR assay	Primerdesign Ltd.	ORF1 ab gene	Nasal, OP swabs, BAL
106.	PhoenixDx® 2019-nCoV	Procomcure Biotech GmbH (Trax Management Services Inc.)	E and RdRp gene	Nasal, NP, OP swabs, BAL
107.	PhoenixDx® SARS-CoV-2 Multiplex	Procomcure Biotech GmbH (Trax Management Services Inc.)	ORF1ab and N genes	NP, OP, AN, MT swabs, BAL
108.	Compass Laboratory Services SARS-CoV2 Assay	Compass Laboratory Services, LLC	ORF 1ab	NP, OP, AN, MT swabs, nasal, NP wash/aspirate
109.	QIAstat-Dx Respiratory SARS-CoV-2 Panel	QIAGEN GmbH	RdRp and E genes	NP swabs
110.	FRL SARS CoV-2 Test	University of Alabama at Birmingham Fungal Reference Lab	N1 gene	Nasal, NP, OP, MT, AN swabs, NP wash/aspirate, nasal aspirate, BAL
111.	DSL COVID-19 Assay	Diagnostic Solutions Laboratory, LLC	N1, N3 and S genes	Nasal, MT, NP, OP swabs, BAL
112.	HealthQuest Esoterics TaqPath SARS-CoV-2 Assay	HealthQuest Esoterics	ORF1ab, N, and S genes	NP, OP, AN, MT nasal swabs, NP wash/aspirate or nasal aspirates, BAL

113.	Clarifi COVID-19 Test Kit	Quadrant Biosciences Inc	RdRp genes	Saliva swabs
114.	Quest SARS-CoV-2 rRT-PCR Kit	Quest Diagnostics Infectious Disease, Inc.	N1 and N3 genes	NP, OP swabs, sputum, tracheal aspirates, BAL
115.	Quest Diagnostics RC COVID-19 +Flu RT-PCR	Quest Diagnostics Infectious Disease Inc	N1 and N3 genes	Nasal swab
116.	Lyra® SARS-CoV-2 Assay	Quidel Corporation	pp1ab (non-structural polyprotein) gene	NP, OP swabs
117.	Solana SARS-CoV-2 Assay	Quidel Corp.	Orf1ab	NP, nasal swabs
118.	New York SARS-CoV-2 Real-time Reverse Transcriptase (RT)-PCR Diagnostic Panel	Wadsworth Center, New York State Department of Public Health's (CDC)	N1 and N2 genes	NP, OP swabs, sputum
119.	Rheonix COVID-19 MDx Assay	Rheonix, Inc.	N1 gene	NP, OP, AN, MT, nasal swabs, nasal wash/aspirate BAL
120.	CDC 2019-nCoV Real-Time RT-PCR Diagnostic Panel (CDC)	Centers for Disease Control and Prevention's (CDC)	N1 and N2 genes	NP, OP swabs, sputum, lower respiratory tract aspirates, BAL, nasal, NP wash/aspirate
121.	Cobas SARS-CoV-2 RT-PCR Kit	Roche Molecular Systems, Inc.	ORF1 a/b	Nasal, NP, OP swabs
122.	CentoFast-SARS-CoV-2 RT-PCR Assay	CENTOGENE US, LLC	E and RdRp genes	OP swabs
123.	Psoma COVID-19 RT Test	Psomagen, Inc.	N1 and N2 genes	Nasal, MT, NP, OP swabs, BAL
124.	Cobas SARS-CoV-2 & Influenza A/B Nucleic Acid Test	Roche Molecular Systems, Inc.	ORF1 a/b and nucleocapsid protein gene with matrix gene of Influenza A, and non structural protein gene of Influenza B	NP, nasal swabs
125.	Novel Coronavirus (2019-nCoV) Nucleic Acid Diagnostic Kit (PCR Fluorescence Probing)	Sansure Bio Tech Inc.	ORF1ab and N genes	NP, OP, AN, MT swabs
126.	ScienCell™ SARS-CoV-2 Coronavirus Real-time RT-PCR (RT-qPCR) Detection Kit	ScienCellTM Research Laboratories	N1 and N2 genes	Nasal, NP, OP swabs, BAL
127.	STANDARD M nCoV Real-Time Detection kit	SD Biosensor, Inc	ORF1ab, E genes	NP, OP, nasal, MT, nasal swabs, sputum

128.	U-TOP™ COVID-19 Detection Kit	Seasun Biomaterials	ORF1ab and N genes	OP, NP, AN, MT, nasal swabs, nasal, NP wash/aspirate, sputum
129.	AQ-TOP COVID-19 Rapid Detection Kit	Seasun Biomaterials	ORF1ab and N gene	OP, NP, AN, MT, nasal swabs, nasal, NP wash/aspirate, sputum
130.	Allplex™ 2019-nCoV Assay kit	Seegene Inc	RdRp, N and E genes	NP, OP, AN, MT swabs, sputum
131.	Sherlock CRISPR SARS-CoV-2 Kit	Sherlock BioSciences, Inc.	ORF1ab and N genes	Nasal NP, OP swab, BAL
132.	Ezplex SARS-CoV-2 G Kit	SML GENETREE Co., Ltd	RdRP and N genes	NP, OP swabs, sputum
133.	Talis One COVID-19 Cartridge Pack	Talis Biomedical Corporation	ORF1ab and N genes	Nasal, MT swabs
134.	ExProbeTM SARS-CoV-2 Testing Kit	TBG Biotechnology Corp	RdRp gene, N and E genes	OP, NP, AN, MT swabs, nasal, NP wash/aspirate, BAL
135.	TaqPath COVID-19 Combo Kit	Thermo Fisher Scientific Inc	ORF1ab, S and N genes	NP, OP, nasal, MT swabs, NP aspirate, BAL
136.	TaqPath COVID-19, FluA, FluB Combo Kit	Thermo Fisher Scientific Inc	ORF1ab, S and N genes	NP and AN swabs
137.	TaqPath™ COVID-19 Pooling Kit	Thermo Fisher Scientific Inc	ORF1ab, S and N genes	NP, OP, AN, MT, nasal swabs
138.	TaqPath COVID-19 FAST PCR Combo Kit 2.0	Thermo Fisher Scientific Inc	N, ORF 1a, and ORF 1b	Saliva
139.	Amplitude™ Solution with TaqPath COVID-19 High Throughput Combo Kit	Thermo Fisher Scientific Inc	ORF1ab, S and N genes	AN swabs
140.	COVID-19 RT-PCR peptide nucleic acid (PNA) Kit	TNS Co., Ltd (Bio TNS)	RdRp and N genes	NP, OP, AN, MT swabs NP wash/aspirates or nasal aspirates, BAL
141.	LifeHope 2019-nCoV Real-Time RT-PCR Diagnostic Panel	LifeHope Labs	N1 and N2 genes	Nasal, MT, NP, OP swabs, BAL
142.	ViroKey™ SARS-CoV-2 RT-PCR Test v2.0	Vela Operations Singapore Pte Ltd	Orf1a and RdRp genes	NP, OP, AN, MT, nasal swabs, nasal or NP aspirates, nasal washes, BAL
143.	SARS-CoV-2 Test Kit (Real-time PCR)	Xiamen Zeesan Biotech Co., Ltd.	ORF1ab and N genes	NP, OP, AN, MT swabs, nasal, NP wash/aspirate, BAL
144.	UCSD RC SARS-CoV-2 Assay	University of California San Diego Health	ORF1ab gene	Nasal, NP, OP, AN, MT swabs
145.	COVID-19 Nucleic Acid RT-PCR Test Kit	ZhuHai Sinochips Bioscience Co., Ltd	ORF1ab and N genes	Nasal, oral swabs

146.	Quick SARS-CoV-2 rRT-PCR Kit	Zymo Research Corp	N genes	Upper and lower respiratory specimen
